# Idiopathic Eosinophilic Cholecystitis in the Setting of Cholelithiasis: A Case Report and Brief Literature Review

**DOI:** 10.7759/cureus.89424

**Published:** 2025-08-05

**Authors:** Smara Sigdel, Stephen Wilson, Elaria Habib, John Diks

**Affiliations:** 1 Pathology, Marshall University Joan C. Edwards School of Medicine, Huntington, USA; 2 Surgery, Marshall University Joan C. Edwards School of Medicine, Huntington, USA

**Keywords:** acalculous cholecystitis, calculous cholecystitis, cholecystectomy, eosinophilic cholecystitis, eosinophils, histopathology

## Abstract

Eosinophilic cholecystitis (EC) is a rare inflammatory condition characterized by prominent eosinophilic infiltration of the gallbladder wall. It often mimics typical acute cholecystitis and is usually diagnosed only after histopathological examination. This case report discusses a 69-year-old woman presenting with symptoms of acute cholecystitis who was later found to have EC. The report highlights the clinical presentation, diagnosis, treatment, and relevant literature on this uncommon condition.

## Introduction

Eosinophilic cholecystitis (EC) is an uncommon variant of cholecystitis, originally identified in 1949 [1]. It is defined by a dense inflammatory infiltrate of >90% eosinophils into the gallbladder wall [2-4]. Though rare, EC remains valuable diagnostically, given that it presents within 0.25% and 6.4% of all cases of gallbladder inflammation [2]. Three large-cohort studies have further solidified the low prevalence of EC [3-5].

EC tends to be more common in women, frequently those between the ages of 35-45, and the clinical presentation is usually indistinguishable from typical calculous cholecystitis [3-5]. Diagnostic symptoms of acute cholecystitis include upper-quadrant pain, positive Murphy’s sign, elevated c-reactive protein (CRP) and leukocytes, and fever [6]. Other common findings include radiation of pain to the right shoulder blade, nausea, vomiting, and exacerbation of pain upon consumption of fatty food [7]. Chronic cholecystitis features similar symptoms, which may be recurrent, with an insidious onset, and/or progressing [7]. Connections to peripheral eosinophilia, allergies, parasitic triggers, and autoimmune diseases have been reported, though diagnoses of EC can be made in their absence [5].

The overall pathogenesis of EC remains uncertain, but Memis et al. propose that its onset is one of two causes: either compromised gallbladder epithelium results in eosinophil infiltration, or systemic eosinophilia manifests as eosinophils within the gallbladder wall with minimal overall damage [5]. Thus, given that EC is unidentifiable clinically, its diagnosis is often made postoperatively [3,4]. EC lies within a spectrum with lympho-eosinophilic cholecystitis, which is defined by an eosinophilic infiltrate of 50-70% [3]. Diagnostic approaches also include defining mucosal versus transmural involvement and chronicity, the latter of which is consistent with the approach to more prevalent forms of cholecystitis [5]. Acutely, cholecystitis progresses from edematous to necrotizing to suppurative; the edematous phase may feature small blood vessels and edema in the subserosa, the necrotizing phase consists of separated necrotic areas across different layers, and finally, during the suppurative phase, leukocytes penetrate necrosed regions to form intramural or peri-cholecystic abscesses [8]. The histopathology of chronic cholecystitis is less defined but may include a thickened gallbladder wall, adhesions, fibrosis, smooth muscle hypertrophy, Rokitansky-Aschoff sinuses, inflammation (first by T lymphocytes, then by plasma cells and histiocytes), metaplasia, and hypertrophy of the muscularis mucosa [7,9].

The infrequency of EC and its clinical similarities to other forms of cholecystitis introduce diagnostic challenges. In this report, we present a 69-year-old woman whose clinical and radiological findings indicated acute cholecystitis, which was later determined to be EC of idiopathic origin following histological analysis. This case highlights the need for further vigilance when diagnosing cholecystitis and supports its definition per the previous literature.

## Case presentation

A 69-year-old woman was transferred from an outside hospital to the emergency department with right upper quadrant (RUQ) abdominal pain. She had experienced intermittent abdominal pain for approximately one week, with progressive worsening. The patient reported an exacerbation of symptoms after consuming burgers the previous day, followed by multiple episodes of non-bloody, non-bilious vomiting. She denied fever, chills, or bowel habit changes. There was no history of similar pain in the past.

Her past medical history included a transient ischemic attack (TIA), for which she was not on anticoagulation, and well-controlled hypertension. She had no prior surgical history. Social history was notable for former tobacco use, and she denied alcohol or illicit drug use.

Initial evaluation at the referring hospital included laboratory studies, ultrasound, and computed tomography (CT) imaging of the abdomen and pelvis. Laboratory evaluation on admission revealed mild leukocytosis with a WBC count of 11.78 × 10³/μL and a neutrophil predominance of 82.3%, suggesting an acute inflammatory process. Absolute neutrophil count was elevated at 9.7 × 10³/μL. Lymphopenia was also present (7.2%, absolute count 0.85 × 10³/μL), while the eosinophil percentage was within the normal range (2.4%). The metabolic panel showed mild hypokalemia (3.2 mEq/L), low serum calcium (8.1 mg/dL), and a slightly elevated fasting glucose (128 mg/dL). Creatinine was low (0.57 mg/dL), with preserved kidney function (eGFR 98 mL/min/1.73 m²). Liver enzymes were within normal limits; however, total bilirubin was elevated (1.8 mg/dL), consistent with biliary obstruction or inflammation. Lipase was low (8 U/L), arguing against pancreatitis. The imaging demonstrated a distended gallbladder with wall thickening, a large gallstone impacted at the neck, and minimal pericholecystic fluid. The imaging and laboratory findings were consistent with the clinical impression of acute calculous cholecystitis. She was treated with intravenous morphine and piperacillin-tazobactam prior to transferring.

**Figure 1:**
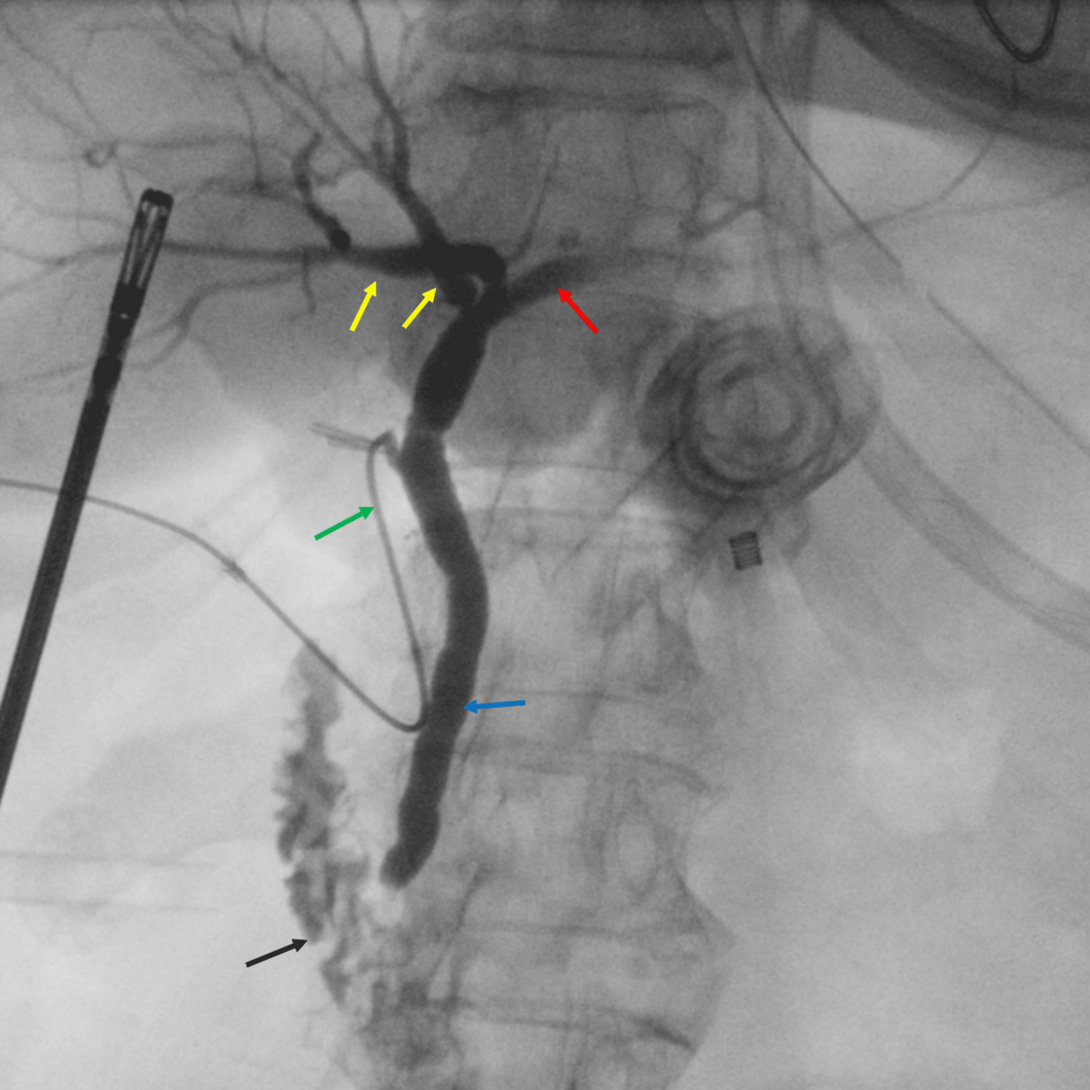
Intraoperative cholangiogram X-ray shows fluoroscopic contrast injection through an angiocatheter (green arrow), demonstrating full opacification of the cystic duct (red arrow), common bile duct (blue arrow), and both hepatic ducts (yellow arrows), with clear flow into the duodenum (black arrow) and no signs of ductal injury.

On arrival, she underwent laparoscopic cholecystectomy with intraoperative cholangiography. The gallbladder was found to be inflamed, thick-walled, and distended. A clip was placed at the infundibulum-cystic duct junction, a ductotomy was made, and an angiocatheter was inserted and secured with a second clip. Fluoroscopy confirmed proper filling of the cystic duct, common bile duct, and right and left hepatic ducts, with good contrast flow into the duodenum and no evidence of ductal injury (Figure 1). The cystic duct appeared long.

**Figure 2:**
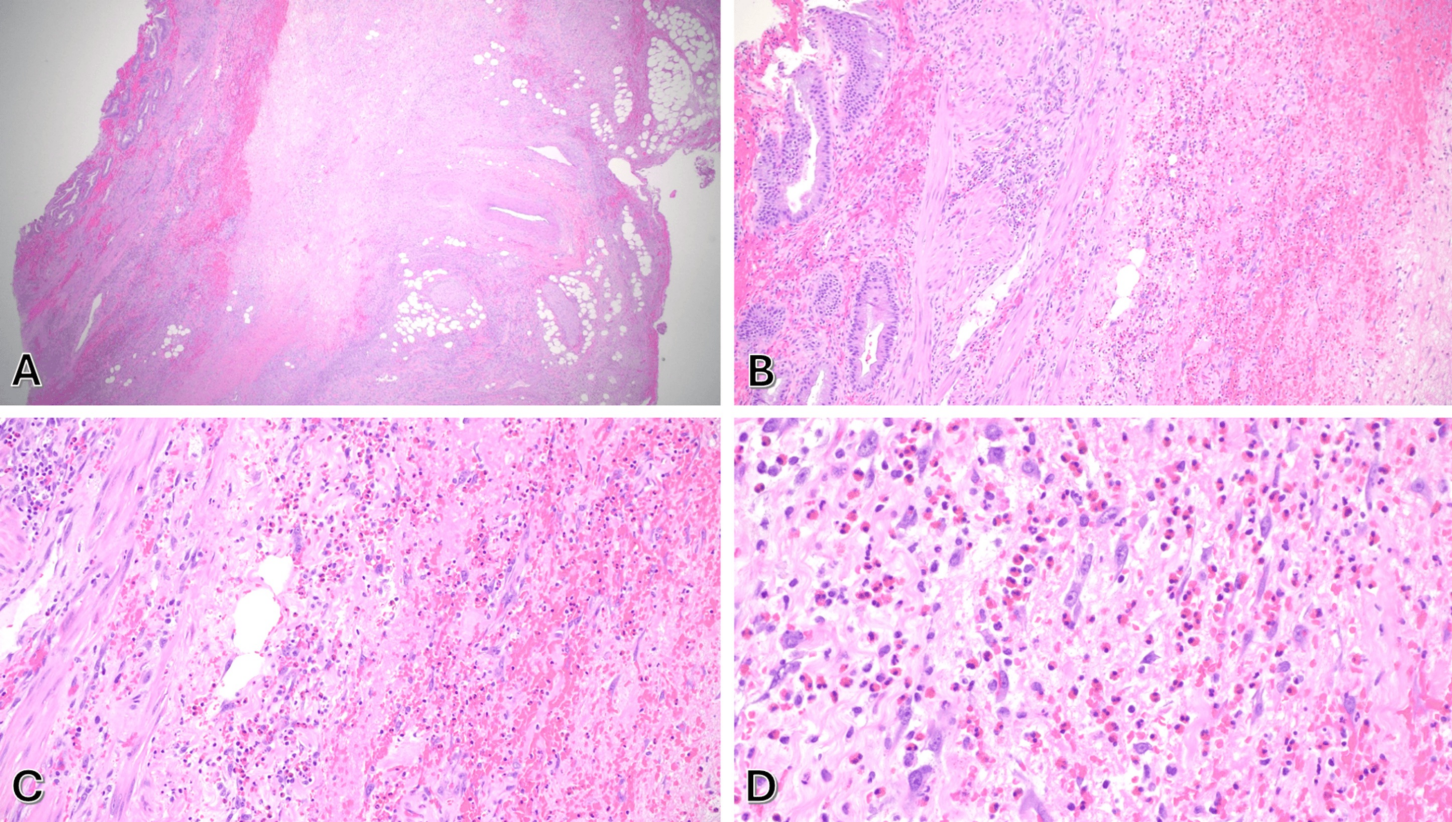
(A) Hematoxylin and eosin (H&E) (20x) showing gall bladder wall thickness up to 1.0 cm with focal congestion and ulceration. (B) H&E (100x) showing transmural inflammation of the gall bladder mucosa and wall. (C-D) H&E (200x & 400x) at higher magnification showing that eosinophils comprise over 90% of the inflammatory cells.

Gross examination of the gallbladder revealed a pink tan to gray-red, slightly disrupted, and roughened serosal surface. The gallbladder measured 7.2 × 3.0 × 2.5 cm. On sectioning, the mucosa appeared red-brown and irregular, with a wall thickness up to 1.0 cm. A 0.5 cm yellow-brown gallstone was present in the neck. Adjacent to the cystic duct margin, a 2.3 cm tan-red structure, consistent with a lymph node, was identified. Histological analysis demonstrated dense transmural infiltration of eosinophils throughout the gallbladder wall, consistent with EC (Figure 2). No evidence of malignancy was seen. The diagnosis was considered idiopathic, as no systemic eosinophilia, parasitic infection, or drug exposure was identified.

The patient's postoperative course was unremarkable. At a two-week follow-up visit, she reported no new symptoms and returned to her usual activities without complications.

## Discussion

Eosinophilic cholecystitis is a rare diagnosis, typically discovered incidentally on histopathologic examination following cholecystectomy for presumed acute cholecystitis. Despite presenting similarly to more typical forms of cholecystitis, the gallbladders of EC patients demonstrate less wall thickening, fibrosis, and overall destruction [5]. In some cases, EC has also been mistaken for metaplasia or resulted in polyps [10]. The condition can present with or without gallstones and does not always correlate with peripheral eosinophilia, as in our case, the eosinophil percentage was normal (2.4%) [3-5].

EC has been discussed sporadically in the literature, with an increase in publications regarding the disease, especially over the last two decades. The reports published demonstrate the diversity of EC etiology and presentation. Although the precise cause of EC remains unclear, reported associations include parasitic infections, allergies, eosinophilic gastroenteritis, hypereosinophilic syndrome (HES), and drug reactions [3-5]. Shah and Shah highlighted a case in which ascariasis resulted in acute cholecystitis, and Motoya et al. drew a connection to a dust mite allergy [11,12]. Additionally, EC has been linked to cholelithiasis, whether it be acute or chronic; histological analysis of patients was also varied and includes metaplasia, eosinophils in various layers, fibrosis, hyperplasia of epithelium or muscle, wall edema, and eroded mucosa [4,5]. Histological features of patients presenting without cholelithiasis, depending on chronic or acute states, similarly include eosinophilia and/or other leukocytes permeating through various layers, mucosal erosion and ulceration, necrotic vasculitis, and edema [5]. In some cases, EC has even been mistaken for cancer or resulted in polyps [10].

Other reports have featured EC co-occurring with pancreatitis, skin rashes, eosinophilic cystitis, cholangitis, eosinophilic gastritis, eosinophilic enteritis, eosinophilic hepatitis, eosinophilic chronic rhinosinusitis, and eosinophilic polyradiculoneuropathy [10,13-18]. In the same breath, many groups have observed HES or eosinophilic granulomatosis with polyangiitis (EGPA) in conjunction with EC, with the latter also known as Churg-Strauss syndrome [10,14,17,19,20]. Of course, as in our case, EC may also be of idiopathic origins [20]. Being that systemic eosinophilic disorders and/or multi-organ inflammation can be involved in EC, it is not surprising that peripheral eosinophilia is relatively common among patients [5]. In such systemic eosinophilic diseases, medical therapy such as corticosteroids may be necessary. For localized EC, treatment is surgical and curative. The recurrence of EC is rare, particularly when the condition is limited to the gallbladder and associated with cholelithiasis.

In the current case, the patient presented with symptoms indistinguishable from acute calculous cholecystitis. Imaging revealed typical features of gallstone-associated inflammation, and management proceeded accordingly. Only after histological examination was the diagnosis of EC established. The absence of systemic eosinophilia and negative clinical history for parasites or drug allergies supports an idiopathic presentation. Differential diagnosis includes acute calculous or acalculous cholecystitis, xanthogranulomatous cholecystitis, eosinophilic gastroenteritis with secondary involvement, and parasitic cholecystitis. EC should be considered in patients with unexplained eosinophilia or systemic eosinophilic syndromes, though it is rarely suspected preoperatively.

## Conclusions

Eosinophilic cholecystitis is a rare but important histologic variant of cholecystitis. It typically mimics acute calculous cholecystitis clinically and radiographically. Definitive diagnosis relies on postoperative pathology. While most cases are idiopathic, clinicians should consider underlying systemic causes, especially in patients with unexplained eosinophilia. Awareness of this entity may aid in the identification of underlying disorders and prevent unnecessary investigations. Given its nonspecific presentation, EC is likely underdiagnosed, and continued reporting of such cases can help clarify its clinical spectrum. Though treatment remains surgical in most instances, recognition of systemic associations may guide further management when needed. As illustrated in this case, histologic examination remains essential, particularly when no other underlying cause is evident.
